# Is there a place for *Bildung* in preparing Religious Education teachers to support and promote epistemic justice in their classrooms?

**DOI:** 10.1007/s40839-022-00187-5

**Published:** 2022-11-18

**Authors:** Alexis Stones, Jo Fraser-Pearce

**Affiliations:** UCL Institute of Education, London, UK

**Keywords:** Epistemic literacy, Teacher education, Big questions, *Bildung*, Epistemic justice, Religious Education

## Abstract

This article draws on an empirical research project in which we explore the roles and understandings of knowledge in Religious Education (RE). Plural understandings of knowledge in schools (and society) lead us to concerns about the relationships between knowledge and social justice. We define epistemic literacy as the capability to recognise, and critically use, different types of knowledge. We also clarify that one’s own relationship with knowledge(s) is significant and is, therefore, important for students and teachers to develop to respond to the epistemically plural RE curriculum and classroom. Drawing on literacy frameworks to identify the need for non-hierarchical conceptualisations of knowledge that include the expert and everyday (Hannam et al., 2020; Shaw, 2019, Vernon 2020), we acknowledge the need for a particular disposition when approaching knowledge about religion and worldviews. Building on the analysis of our empirical study and subsequent developments of epistemic literacy, we revisit the notion of epistemic justice (Fricker, 2007) and present a theoretical justification for the experiential preparation of teachers that draws on Biesta’s (2002) reformed *Bildung* of encounter and Rawls’ “veil of ignorance” (Rawls, 2005).

What emerges from these reflections on the future of *Bildung* is, therefore, an image of a learning society conceived as a society in which the real encounters with who and what is other are a constant and continuous possibility.

(Biesta, 2002, p. 350)

## Introduction

Building on a study in which we concluded that epistemic literacy might challenge reductive, misconceived and polarising ways that different kinds of knowledge are handled in school Religious Education (RE) (Pearce et al., 2021), we embarked on an empirical research project (funded by Templeton World Charity Foundation) concerned with the development of epistemic literacy in RE. Soon into the analysis of our data we recognised the significance of Miranda Fricker’s “epistemic injustice” (Fricker, 2007) for our findings. Following classroom observations and interviews with teachers and Key Stage 3 students (ages 11–14), it is our contention that some ways of defining and handling knowledge in RE classrooms contribute to epistemic inequality between young people. In Stones & Fraser-Pearce (2021), we distinguish between “epistemic haves and have nots”. One of the key identifiers of the latter is the (often unquestioning) credibility given to opinion as reliable knowledge. This seems largely underpinned by students’ strong commitment to respecting the opinions of others - respect being interpreted by most students to mean ‘uncritical acceptance’. Conversely, the “epistemic haves” — the minority of students we spoke to — emphasise the importance of engaging critically with knowledge claims.

Populist perspectives, a proliferation of media platforms, ‘bots’ of Artificial Intelligence disguised as humans, and advertisements seemingly identical to news stories (Wineberg and McGrew, 2016), all set the scene for an epistemic crisis. We draw on Wolfgang Klafki’s pedagogical frameworks of *Categorial Bildung* and “epoch-typical problems” (Klafki, 1995) to recognise the educational significance of this crisis and the urgency of epistemic justice, broadly speaking. Having established that epistemic justice, and therefore epistemic literacy, is a matter of social justice (Stones & Fraser-Pearce, 2021), it follows that teachers have a responsibility to support and promote epistemic justice for their students. A question arising from this, and the main concern of this paper, is how we prepare RE teachers to do this?

We attempt to answer four questions in this paper, with a section devoted to each:

- *What is epistemic injustice, and what does it look like in secondary (Key Stage 3) RE?* Here we include an account of our methodology.
- *What is epistemic literacy, and how might it contribute to epistemic justice?*
- *What approaches to teaching and learning hinder, and what approaches might promote and support, the epistemic literacy of learners in RE?*
- *How can we prepare RE teachers to teach for epistemic literacy and justice?*

Despite current enthusiasm for disciplinary knowledge in RE at a curricular and pedagogical level, the disciplinary knowledge forms of RE only partially contribute to the complex epistemic landscape of the subject and its enacted curriculum. In our roles as teacher educators, members of a teacher education community, and former RE teachers, this is of great concern to us. An informative scenario that one of us recalls from teaching RE involved a student entering the RE classroom exclaiming “I don’t believe in RE, I believe in science”. While this may seem a caricature, our recently collected data reflects both this conflation of terms associated with each subject (one does not typically use the term ‘belief’ in science) and a perceived polarisation of different disciplines and knowledge forms. The student teachers of RE, whom we are tasked with preparing, also come with (a range of) preconceptions about what the subject involves in epistemological terms (see Commission on Religious Education 2018, Ofsted 2021, Norfolk Locally Agreed Syllabus 2019).

The limitations of the compatible/incompatible model of relationships between religion and science seen in the literature (Polkinghorne, 1986; Barbour, 1990) and commonly in RE curricula, epitomise our concerns about how knowledge is often (although not always) handled in a reductive and polarising way in RE. We began a research project that asks, *What distinctive role, if any, can RE contribute to the development of epistemic literacy regarding the relationships between religion and science?;* and have found ourselves engaged in a more complex and, seemingly, increasingly urgent discussion around knowledge and justice.

In 2022 the contestations of knowledge and expertise are in the public discourse in renewed contexts produced by some of the effects of Covid 19. Former Chancellor and one of the final two candidates for Prime Minister in 2022, Rishi Sunak, reached headlines in a recent interview in which he expressed regret over scientific experts being too “empowered” in relation to lockdown measures during the pandemic (Nelson, 2022), whilst epidemiologists refute his claims saying their expertise and advice were ignored in the early stages of the pandemic (Nicholson, 2022). Following teachers’ assessments in the absence of public examinations, evidence has emerged that 'A’ Level results from private schools were proportionately graded higher by teachers than at non-private schools. Aside from accusations that teachers were over-estimating students’ grades and ‘gaming the system’ (Henry, 2022), the links between epistemic authority and justice are once more (that is, since our heavy reliance on experts during the pandemic) in the public eye. Disputation of knowledge and expertise has had, and continues to have, critical effects on health, lives and livelihoods - an effect of the epistemic injustices suffered by the population.

## What is epistemic injustice, and what does it look like in secondary (Key Stage 3) RE?

Fricker describes epistemic injustice as the wronging of someone “specifically in their capacity as a knower” (Fricker, 2007, p.1). There is good reason to be concerned that RE in some schools in England contributes to epistemic injustice (Stones & Fraser-Pearce, 2021). This section begins with an exploration of Fricker’s epistemic injustice. Following a brief account of our methodology, we draw on our empirical study to paint a picture of epistemic injustice in Key Stage 3 RE. It is important to say that we found plenty of good practice which enabled epistemic literacy and justice in schools. Given the focus of this paper, however, we have presented examples from our data which illustrate epistemic injustice.

Fricker identifies “social power” as the unjust effects of a structural monopoly of epistemic authority experienced at individual levels as either an excess or deficit of credibility. This disparity is linked to perceived “identity power” which operationalises “power that depends in some significant degree upon such shared imaginative conceptions of social identity” (Fricker, 2007, p.130). It is not difficult to imagine the demonstration of this in the teacher’s epistemic power and the implicit communal monopoly of those who project a similar identity, perhaps through language or traits that suggest confidence.

Fricker goes on to recognise the significance of “hermeneutical injustice” as result of “hermeneutical marginalisation” in which a minority group or individuals do not contribute to meaning making in the epistemic sense. This can be at a local, global, individual and group level. This marginalisation is furthered by “testimonial injustice” and “pre-emptive testimonial injustice”. The former describes the event of someone not being believed due to prejudice, and the latter is when a person (or people) who is deemed untrustworthy is not even asked. “Testimonial silencing” is a self-silencing that results from the awareness of one’s lack of credibility due to the injustices described here. The lack of power and freedom evoked in Foucauldian incarceration imagery of the internalised panoptic gaze resonates with this self-imposed exclusion (as a result of explicit exclusion) that denies epistemic justice to individuals and communities.

Like many areas and aspects of formal and informal education, RE is susceptible to epistemic injustice through its policy, curriculum, pedagogies, disciplines, teachers, students, school structure and subject status, time allocation, and perceived nature(s) and purpose(s) of the subject. Consideration of each of these elements is beyond the scope of this article; however, it is relevant to our specific research concerns to consider what voices, histories and ontologies are excluded from the RE curriculum. James Holt’s (2019) work acknowledges the importance of including religious and non-religious traditions in the RE curriculum that extend beyond the usual “big six” religions, with attention to reflect the traditions of the students in one’s classroom. But is this enough?

Assuming RE moves towards a worldviews and disciplinary-focused curriculum, attention must be paid to the possibility of epistemic injustice. Some discussions around knowledge claim that disciplines have power and potential for social justice through their facilitation to imagine the not yet imagined (Young and Muller 2010, Deng 2021). The disciplines associated with RE, however, are prone to a homogenisation of discourse in the curriculum and classroom that may exclude diverse perspectives. Theology, Philosophy and Social and Human Sciences are held by advocates of a religion and worldviews curriculum to maintain academic rigour for the subject (Commission on Religious Education, 2018, Ofsted 2021, Norfolk Locally Agreed Syllabus 2019). In their analysis of values in the curriculum, Mitchell & Stones (2022) argue that these ‘parent disciplines’, founded in the academy, are built on colonial foundations and values that sought to categorise, order and examine through a Christian, heterosexual, European, male lens.

In our study we wanted to find out how knowledge is used and understood by students and teachers in RE classrooms. In order to do so we conducted interviews with RE teachers and Key Stage 3 students, observations of Key Stage 3 RE lessons, and an online RE teacher survey. In this paper, we draw upon data collected in interviews and observations. We visited eight contrasting schools around England and interviewed about three teachers, and five or six student groups, in each school—amounting to 20 teachers and 36 groups of students. Participating schools included: rural, suburban and urban schools; boys, girls and co-educational schools; schools of religious character and ‘common schools’; independent, grammar and comprehensive schools; and schools from a range of English counties.

We were particularly concerned with how knowledge is handled in RE in relation to the kinds of big questions (often, but not always, in connection with science and religion) in the curriculum. As such, all interviews began by presenting participants with examples of what we consider to be big questions, such as:

Why did the universe begin?

Is there life after death?

How do we know what being good or bad is?

How do we know whether something is right or wrong?

How do you know if something is true or false?

With the benefit of hindsight, we now notice the easy fit of the latter three questions with the polarisation with which we have become concerned. We would express these in different ways now, resisting the draw of the simple and coherent (Lombrozo, 2007) and the notion of an either/or explanatory space (Preston & Epley, 2009). Nevertheless, our initial expression is perhaps a symptom of the ubiquity of polarisation, and/or a habit nurtured by our own school education. We are surmising here but it’s worth noting, firstly, that either might make for hard habits for teachers to break; and secondly, that this emphasises the importance of recognising this as we prepare RE teachers.

We began interviews by focusing on the nature of big questions. On turning to knowledge, we asked students:

What kind or kinds of knowledge would you need to answer big questions?

How would you know you are using the right kind of knowledge? (Knowledge you could rely on/trust?)

We asked teachers:

What kind or kinds of knowledge would/do students need to answer big questions?

How would they know if they are using the right kind of knowledge? (Knowledge they could rely on/trust?)

We did not present an understanding of ‘knowledge’ to participants (even when they asked), as we wanted to find out about their understandings and interpretations.

As we have argued elsewhere (Stones & Fraser-Pearce, 2021), we were first alerted to the possibility of epistemic inequities between classrooms when we found that some students and a few teachers conflate knowledge and opinion. Relevant excerpts from interviews are presented in our 2021 paper. Some bear repeating here, with the first few demonstrating epistemic disadvantage and the latter advantage – these participants might be viewed, respectively as the epistemic have-nots and the epistemic haves:

Examples of epistemically disadvantaged students:

RE is there to teach you to respect other religions and their beliefs.

Student 1: “So [in] science your opinion [that the earth is flat] would be wrong, but no opinion is actually wrong.” Student 2: “... unless it is, like ... an opinion about someone, like not a very nice opinion.”

Maths doesn’t have your opinion, it’s what’s right and wrong, the answer is right or wrong. But in RE there is no right or wrong, it’s your opinion.

Because everyone’s allowed to have like their own opinion. So, I guess the only real knowledge you need [to answer big questions] is your own opinion.

We identified some students as displaying epistemic advantage:

Opinions are what you believe, knowledge is what you’ve been taught, and facts are what is actually true.

How to throw a normal jab, right, and an uppercut... [T]hat’s the knowledge on boxing... [H]e trained more, and he knew more about boxing and what to do. So his knowledge helped him.

I’ve definitely thought about [big questions] a lot more, since doing them in RS... I always used to have my own answer and think that nothing could disprove it... But now I hear lots of evidence, it’s really hard to make a decision now.

If data from interviews tell us what epistemic injustice sounds like, then it follows that observations can tell us what it looks like in the classroom. We know from our experience and research that the following scenarios are relatively common in RE classrooms: approximately 30 young teenagers are presented with a big question or issue in the abstract and are asked to choose (often publicly) between two options – agree/disagree, for/against, right/wrong. In our study, for which we personally conducted all classroom observations, we observed examples of these activities where students had to publicly indicate whether they were “for” or “against” an issue relating to medical ethics by standing in the “for” or “against” line. Other adaptations included students being asked to vote (with little or no discussion) on which arguments should “win”. These kinds of activities contribute to epistemic injustice, or do wrong to students in their capacities as knowers (Fricker, 2007), as they neither encourage nor enable sufficiently nuanced or sensitive engagement with the complexities and realities of the issues at hand; they promote reductive evaluation and uninformed decision-making. The approaches discussed here also contribute to a performative dimension to decision-making with potentially divisive consequences that can overshadow authentic engagement. In turn, they fail to prepare students to engage appropriately with issues and questions they may face in their futures.

Some of the activities in which students were encouraged to engage with differing positions did little to enable appropriate engagement, as indicated in the excerpt below, from our fieldnotes:

Lesson title: ‘Miracles’...

The teacher introduces the lesson: “Some of you might agree with miracles, and some of you might be against them, and that’s okay.”...

Teacher asks class: “What is a miracle?”. Some students offer definitions (e.g., “the impossible happens”) and some examples (e.g., “when you pray for something to happen and then it does”, and “it’s a miracle Mum didn’t burn the turkey on Christmas Day”). Neither teacher nor students distinguish between definitions and examples, or between different kinds of examples...

The teacher walks around the room whilst students are on task writing about whether or not miracles happen. He reminds students to include “both views”. Again, he is suggesting that there are two polar views, one or the other...

(Fieldnotes, Year 7 RE lesson “a”)

There are a few ways in which this lesson could contribute to epistemic injustice. Firstly, in the lack of precision of the language used in the introduction to the lesson. ‘Agree’ is usually contrasted with ‘disagree’, and ‘for’ with ‘against’. It is more usual to refer to ‘believing in’, rather than ‘agreeing with’ miracles, and it is not clear what it might mean to ‘be against’ miracles. We recognise that the students would have understood what this teacher meant. Nevertheless, we do not think we are being pedantic here; as we elaborate in the next section, literacy matters. Secondly, without curation, the array of responses to the question of what constitutes a miracle highlights the need to establish that there are different interpretations of miracles for an informed discussion to take place. By making the students’ interpretations explicit, the teacher could have supported students in understanding there are a range of ways of thinking about and answering this question (and, therefore, other questions). Indeed, not to do so permits epistemic injustice as students work towards conclusions without an understanding of what frames of reference (perhaps literal, symbolic, theological, or an idiom) are in play. Finally, as we note in the excerpt itself, students are encouraged to think in terms of polar views.

In this section, we have drawn on our data to illustrate what epistemic injustice looks and sounds like. To do so, we have mainly focused on examples of epistemic disadvantage. Our data also includes examples of epistemic advantage. Indeed, it is the nurture of epistemic advantage for some (the haves) and of epistemic disadvantage for others (the have nots) which make this a justice issue. Above we note that literacy matters. In the next section we elaborate by explaining what we mean by epistemic literacy and why we think it might contribute to epistemic justice.

## What is epistemic literacy, and how might it contribute to epistemic justice?

The adjective ‘epistemic’ means ‘relating to knowledge and knowing’. UNESCO define ‘literacy’ “as a means of identification, understanding, interpretation, creation, and communication in an increasingly digital, text-mediated, information-rich and fast-changing world.” (https://en.unesco.org/themes/literacy).

As Stordy discusses in his taxonomy of literacies (2015), the practice of qualifying the noun ‘literacy’ with an adjective can be seen in a range of fields:

The 1980s witnessed the fracturing of literacy into various subject literacies. These essentially meant competence or proficiency in some associated subject area... For example, being maths literate or environmentally literate meant that a person knew how to operate the language of the subject well enough to make sense of it. It also saw the origins of literacies ... that attempted to encapsulate ... skills and competencies... For example, the concept of computer literacy became increasingly prevalent to encapsulate the skills and competences necessary to effectively use computers...

(Stordy 2015, 457)

Following Stordy (2015) and building upon UNESCO, we define ‘epistemic literacy’ as:

Competency and proficiency in the identification, interpretation, understanding, questioning, navigation and communication of knowledge.

Our shorthand for this is ‘knowing well’.

There are three main discourses that we draw on to clarify the meaning and functionality of epistemic literacy: religious literacy, epistemic switching and capabilities. Religious literacy, according to Hannam et al., (2020), emphasises the importance of the educator’s role in including language and tradition that are beyond the “dominant” language and discourse. Shaw’s formulation of “religion and worldview literacy” (2019), on the other hand, identifies the need for the educator’s and student’s reflexivity, and the development of a disposition of tact and insight informed by (1) knowledge of the actual religious and non-religious landscape, and (2) a nuanced grasp of what the category of religion/worldview entails.

As a response to the research and development of “epistemic insight” by Billingsley et al., (2013) which opens up discussions around the different types of knowledge across subject disciplines, we recognise the importance of the work of Gottlieb and Wineburg (2012). They make the case for “epistemic switching” by citing examples of when their participants unconsciously “switched” between their religious, social and academic identities to respond to stimuli. Their recommendations stress:

The idea that epistemology and identity can affect each other not only vertically (by providing the cognitive conditions for holding particular beliefs about knowledge or the self) but also horizontally (by triggering different kinds of identification and belonging as the context shifts) has potentially radical implications for theories of both identity and epistemology... relations between these two seemingly distinct constructs may be much closer than has been previously assumed.

(Gottlieb and Wineburg 2012: 117–118)

‘Epistemic literacy’ responds to the authors’ invitation to develop “theories of learning [that] extend beyond the “merely” academic to touch on practical concerns about how to educate real people about things that matter” (ibid. 118).

Our account of epistemic literacy also owes much to Michael Young and colleagues’ discussions around Powerful Knowledge (Young & Muller, 2010; Young & Lambert, 2014; Deng, 2021) which, broadly speaking, describe the notion that certain disciplinary kinds of knowledge are ‘powerful’ in the sense that they take people beyond their everyday knowledge. Thus, schools and disciplines should provide epistemic environments in which expert knowledge can be encountered and developed as a matter of social justice.

As we have mentioned in previous publications, the “capabilities approach”, as conceived by Nussbaum and Sen (Nussbaum, 2011; Sen, 1999) and incorporated into the Geocapabilities project (Young & Lambert, 2014, Lambert et al., 2015; www.geocapabilities.org), is also crucial to our understanding of the role of epistemic literacy and its relationship with epistemic justice. In the same way as Fricker sees epistemic justice as a capability (Fricker, 2007), epistemic literacy is necessary for the handling of knowledges during school years and beyond. Furthermore, epistemic literacy relates strongly to Nussbaum’s capabilities regarding health and the ability to make informed judgements. This relationship is reflected in concerns over sources of knowledge: conspiracy theories, ‘fake news’ in mainstream media (Stones & Fraser-Pearce, 2021), as well as contestation of expert advice, as previously mentioned.

Vernon reconciles the tension between a constructivist Vygotskian approach and the distinction of expert knowledge in her proposal that acknowledges the significance of the ‘epistemic self’. She calls educators to implement a dialectical and iterative approach to everyday and expert knowledge. Vernon’s proposal recognises the epistemic world of learners, and the conceptual world of subjects; she sees them as generative only if educators are able to bring these into relationship (Vernon, 2020). This echoes our concern that epistemic literacy includes the capability to competently and proficiently navigate a range of diverse claims to knowledges in order for epistemic justice to emerge.

## What approaches to teaching and learning hinder, and what approaches might promote and support, the epistemic literacy of learners in RE?

Our data briefly discussed above indicate that students’ and teachers’ conflation of terms suggest a confusion relating to distinctions between knowledge, belief and opinion. This constitutes a deficit of epistemic literacy. We also saw and heard examples of some teachers offering simplistic taxonomies of relationships between religion and science that loosely cohere with Barbour’s conflict, harmony, integration, independence model (Barbour, 1990) and offer binary *either* religion *or* science understandings. The following is an excerpt from our fieldnotes written during our observation of a year 7 RE lesson.

Lesson title: ‘Creation: science or religion?’...

Learning objective: ‘To understand different opinions on how the world was made’...

Teacher’s verbal instruction for starter activity: ‘On your table, can you think of a logical explanation of how the world was made? You’ve got 90 seconds’. Student responses included: ‘the big bang’, ‘I don’t know’, and ‘Jesus’...

The teacher moves on to the next slide which listed the ‘different viewpoints’ they would be looking at in the lesson: ‘Big Bang Theory’, ‘Hindu creation story’, ‘Genesis’. The teacher describes Genesis as ‘what Christians believe’ and follows this up with ‘you can decide what you believe; you might want to take bits from all’. She then plays the three YouTube videos in turn. The ‘Big Bang’ video is difficult to access, complicated and serious in tone. The two religious accounts are more accessible, with the account of Genesis (1) being very simplistic – quite babyish in fact. The two religious accounts posed no challenge to students, whereas the scientific account was inaccessible...

Following the input of the videos, the next slide included the main task of the lesson: ‘In pairs, create a presentation on how you think the world was made’. The teacher verbally elaborated: ‘Come up with a sensible explanation of how the world was made’...

Towards the end of the lesson, students present their explanations in pairs. This consists in them showing pictures they have created, accompanied by brief statements. For example:

- Student 1: ‘The world was made from two asteroids crashing together.’
- Student 2: ‘God started everything then the space things happened.’...
- Teacher: ‘So, a combination of religion and science?’
- Student 2: ‘Yes.’
- Student 3: ‘We think the world was created by God.’ [These students then quote part of Genesis account.]
- Student 4: ‘We think God created the world.’ [Student’s quote from Genesis.]
- Teacher: ‘So you think that it’s purely what’s in the Bible? No big bang or anything?’
- (Fieldnotes, Year 7 RE lesson ‘b’)

The specific ways in which we think this lesson hinders epistemic literacy point to the ways in which it could be promoted and supported. The imprecise language which, for example, suggests that science might explain ‘creation’ or that different viewpoints constitute ‘opinions’ presents a hindrance. Our definition of epistemic literacy requires competent use of language and this requires precision. The lesson materials present science as (too) complex and religion as simplistic. Both religion and science were presented in reductive terms and mostly as binary opposites.

It is difficult to see how students were enabled to critique or move beyond their everyday knowledge they had before the lesson (this was the first lesson on science and religion). In so far as we saw, they were not given the opportunity to develop any epistemic literacy to support their grasp and navigation of the different knowledge structures, methods and criteria that scientific and religious explanations draw on. Students’ voices and testimony were not heard, and hermeneutic justice was replaced with a curated and reductive representation of scientific and religious explanations aimed at students choosing one explanation rather than problematising its polarising simplicity.

In addition to the problem of polarisation, we also draw on Smith et al., (2018) who point to the problem the ‘sanitization’ of religion in RE for the instrumental purpose of community cohesion. Rather, students should be given opportunities to develop an informed religious literacy that is appropriate for religion as a “multifarious, complex, social phenomena” (Smith et al., 2018, p. 1). Smith et al. refer to Panjwani and Revell’s (2018) observation of educational ‘essentialisation’ of religion that reifies the abstract idea of, for example, a Muslim through a constructed idea of a member of Islam, as if there were only one type of Muslim. To relate this more explicitly to epistemic literacy, we hold that sanitization and essentialisation of religion remove the opportunity to develop understandings of different kinds of knowledge that inform the complexities of affiliative, heritage-related, and intersectional identities. So, when teachers, students or peers demonstrate religious belief or expression that does not correspond to the sanitized or essentialised version, there is no literacy or meta-knowledge to make sense of this. Furthermore, the complexities of *one’s own* perspective must be acknowledged through a reflexive epistemic literacy that asks where and how we form our beliefs, views and knowledge claims.

We posit that RE should foster within students and teachers care for epistemic justice that can be achieved through the promotion of epistemic literacy. If the complexities of epistemic (in)justice, according to Fricker, are made explicit to teachers and students, then they will see how testimony and hermeneutic justice require epistemic literacy for an epistemically just classroom.

We acknowledge, however, that there are objections about subjects ‘adopting’ aims. Standish (2009) holds that subjects should not be used for good causes. He is wary, for example, of geographical knowledge being diluted as a result of aims associated with global perspectives, however laudable the aims, due to moral authoritarianism. Hussain (2018) argues that RE should dispense with its responsibility for community cohesion as this instrumentalisation is at the expense of the subject’s academic rigour. In response to this perceived dichotomy of pro-social aims *or* academic rigour, following Deng (2020, 2021, 2022), we have turned to German and Nordic understandings *Bildung* to seek out the moral and intellectual ‘powers’ of RE (see Deng 2020) through epistemic literacy.

We have articulated elsewhere that the ethical underpinning of epistemic literacy (due to its relationship to justice) is helpfully expressed within the pedagogical and ethical structure of *Bildung* (Stones and Fraser-Pearce 2022). Through collaboration and exchange as part of an international network with University College London, Karstad and Helsinki Universities (KOSS network, funded by the Swedish Research Council), we have developed epistemic literacy further as a proposed aim for RE with implications for teacher education grounded in *Bildung*, both for the pupil *and* teacher.

*Bildung* (loosely translated as formation) is deemed to have foundations in Classical Greek and Roman education, humanism, the European Enlightenment and modern liberal education; it is an education espousing the idea that education creates order on oneself, one’s relations to the world and thus leads to responsibility (Klafki, 1995). This ‘ordering’ and responsibility are considered to emerge from a relationship between the intellectual and moral aspects, for which the academic disciplines are a resource and vehicle (Deng, 2018). Klafki’s (1995) own development of *Bildung* deconstructed the educational concept to distinguish the content (what he refers to as *material Bildung*) and the presentation or pedagogical interpretations of the content that students can relate to *(formal Bildung*). The teacher’s skill lies in their ability to relate the content to the pedagogy and with specific understanding of what Klafki calls ‘epoch-typical problems’, or contemporary challenges such as war, famine, inequality and social injustice and, as we have previously stated, the epistemic crisis of our time. Readers may (justifiably) see this as another iteration of the familiar relationship between curriculum/knowledge and pedagogy, but we wish to draw on *Bildung* further for the purposes of promoting epistemic literacy as an aim for RE *and* RE teacher education for the purposes of epistemic justice.

We propose that an urgent ‘epoch-typical problem’ for RE, such as Klafki identifies, is the epistemic plurality, lack of epistemic literacy and epistemic injustice among some students (and teachers) that our empirical study suggests. In our aforementioned work (Stones and Fraser-Pearce 2022), we make the case for epistemic literacy as a crucial tool for teachers to deconstruct the transformations, or ‘recontextualization’ (see Bernstein 2000), of pedagogical content ranging from curriculum to textbooks and resources. The teacher’s development of her own epistemic literacy allows her to see the epistemological and normative workings of pedagogical content in addition to being aware of her own epistemic biases, preference and blind spots that Gottlieb and Wineburg’s (2012) study suggests.

Our argument here now turns to the RE teacher’s *Bildung* and we propose a justification for an experiential approach to developing the teacher’s epistemic literacy that aims to evoke and address the challenges of epistemic justice. Furthermore, the justification attempts to cultivate care and concern for epistemic justice as an alternative to competing purposes and ideologies of education that Giroux warns are steeped in neoliberal priorities (see Giroux 2020).

## How can we prepare RE teachers to teach for epistemic literacy and justice?

In this section we argue that, as epistemic literacy is twofold, it requires a proficiency in understanding the nature of knowledge that is both external and internal to the self. It is crucially reflexive, and therefore an RE teacher’s professional development demands reflexivity to create awareness of one’s own epistemic tendencies or order to develop a level of proficiency that we outline in our definition of epistemic literacy.

Following our identification of epistemic injustice as an example of Klafki’s ‘epoch-typical problems’, we have found Biesta’s (2002) more recent reflections on *Bildung* helpful in addressing the issue of hegemonic, disciplinary and rational approaches to ‘big questions’ in RE that devalue nuance and discomfort in favour of filling an ‘explanatory space’. Biesta’s critique highlights the problem of Kantian Enlightenment developments of *Bildung* aimed at “rational autonomy” for democratic citizens and identifies a flaw in *Bildung* that is not suited to the needs of plural society. Indeed, rational autonomy is oxymoronic if the criteria for rationality are not one’s own. As fields of education grapple with the epistemic demands of post-coloniality, rational autonomy is no longer sacrosanct.

Biesta’s concern with the lack of distinction between ‘diversity’ and ‘difference’ identifies the issue of diversity implying “a collection of variations that have a similar ground or origin…[meaning that] we are all basically “the same” and that our differences are “merely cultural” (Biesta, 2002, p.346). Smith et al’s (2018) observation of the challenges created by a community cohesion-driven sanitization of religions would certainly echo Biesta’s distinction. Biesta warns that “[t]hinking about plurality in terms of difference is…a way not to mistake the part for the whole…[and thus is] one way to take democracy seriously” (ibid. 347). Rationality cannot be feasibly considered a neutral position, and rather constitutes “but one tradition…[and] relocates the rational life in the world of difference itself” (ibid.). Biesta posits that actual encounter with difference through meeting other people is the only way for meaningful *Bildung* to occur.

Following Biesta’s theoretical grounds for a reformed *Bildung* of difference and encounter, and Klafki’s *categorial Bildung* in which content and pedagogy are brought into relationship through ‘epoch-typical problems’ (which we have attributed to epistemic injustice as a result of epistemic pluralism without epistemic literacy), we turn to the notion of epistemic literacy and epistemic justice as capabilities in the sense that they relate to one’s epistemic rights. Biesta cautions against the normativity of rationality and an imagined totality which corresponds to no-one. This continues our concern that deliberation over epistemic justice can too easily exclude everything other than the rational perspective. Indeed, Nussbaum’s definitive list of capabilities (Nussbaum, 2011) has been criticised for being the result of consensus and therefore “the end point of such discussions” which hide alternative views (Stewart, 2013, p.157). We are also mindful of Stewart’s observation that Nussbaum’s capabilities are too individualist and subjective to be a convincing and universal theory of justice. We are faced with the compelling case for encounter as the only way to know difference and to know one’s own difference, as Biesta proposes. It goes without saying that the practicalities of encountering the amount of people necessary to experience plurality for oneself (as opposed to a series of curated perspectives for the purposes of presenting ‘diverse’ points of view) is more of an ideal than a possibility. Indeed, Biesta’s proposal reaches beyond the timeframe of a school day and school life and is perhaps more akin to an ethic than an event:

What emerges from these reflections on the future of *Bildung* is, therefore, an image of a learning society conceived as a society in which the real encounters with who and what is other are a constant and continuous possibility.

(Biesta, 2002, p.350)

How might we, as teacher educators, translate this possibility into the preparation of teachers, which is time-based and time limited? Although the purview of this paper does now allow a detailed exploration of pedagogical possibilities, readers might consider the pertinence of Rawls’ theory of justice and “veil of ignorance” (Rawls, 2005) as a response to Biesta’s invitation.

Emerging from a socio-political context of the 1950s onwards and characterised by post- and ongoing war, migration, discrimination, protest, inequality and social unrest, Rawls asked what justice is, or could be. His theory is a challenge to the utilitarian politics that had, and still, dominated and which, by definition, did not include the minority (see Rawls 2005). His “veil of ignorance” is a thought experiment in which one does not know who one might be in the effort to construct the rules and rights of a just society. We are certainly not the first to consider the value of Rawls’ thought experiment when looking at the place of religion in schools (Moulin & Robson, 2012; Cruden, 2015) and here we suggest that a Rawlsian theory of justice, that the veil evokes, might also be usefully applied to epistemic justice.

The veil can be understood as a metaphor for the epistemic rules and rights of others. To recall the theory of ‘epistemic switching’, knowing is directly linked to our identity or identities; therefore, by imagining another one might have the possibility of imagining their epistemic perspective and subsequent needs for epistemic justice to occur. The metaphor can be enacted in a curriculum of teacher preparation and applied in various contexts to encounter the epistemic positions of others. Individual and group reflections on this process may also reveal one’s own subjective position, and one’s assumptions about others, with more nuanced understanding. Perhaps this corresponds to a task of life-long journey of self-knowledge that is beyond the scope of an RE teacher education. We recognise, however, the role of the ideal here. Following Biesta’s (2002) *Bildung* of encounter, an ideal correlates with an appropriate ethic that responds to one’s responsibility for an epistemically just classroom as “a constant and continuous possibility” (Biesta, 2002: 350). Another stage of the thought experiment could entail student teachers imagining how their students might respond to the veil experiment. ‘Putting on the veil’ when planning and teaching, would serve as a metaphor for the teacher never being certain what epistemic dimensions are being enacted or not enacted by students in a given RE lesson (or individual moment) that can account for epistemic differences among students.

## Closing words

In Winch et al.’s (2015) call for the development of teachers’ professional knowledge, the authors identify “a moral as well as a practical dimension in paying attention to the values that inform their practice, identifying certain goals rather than others, and in the attitudes they adopt towards the particular students they teach” (Winch et al., 2015: 205). We posit that this moral and practical development emerges as a result of experiential and reflexive processes that potentially lead to epistemic literacy for the purposes of epistemic justice to highlight “complementarities between practical, technical and theoretical knowledge” (ibid.). An approach to teacher preparation that develops epistemic literacy would incorporate these three forms of knowledge and could be targeted by harnessing Biesta’s aspirations through participation in Rawls’ thought experiment as we have described.

While Biesta’s (2002) reformed *Bildung* presents the case for the importance of encounter with difference, we note there is room for an experiential and complimentary approach that Rawls’ “veil of ignorance” provides. As Winch et al., (2015) propose, teacher education should be simultaneously theoretical, practical and technical, while maintaining the inclusion of values and moral consideration of the students being taught. We argue that a combination of these aspects and justifications discussed here potentially contributes to a holistic and subjective teacher education experience. This responds to the epistemic demands of RE, academically, ethically and personally, and the need for personal transformation through reflexivity during a teacher’s preparation. Such a preparation should provide teachers with the tools to develop their own, and promote students’, epistemic literacy for the purposes of an epistemically just curriculum and classroom.
